# Molecular and functional heterogeneity of early postnatal porcine satellite cell populations is associated with bioenergetic profile

**DOI:** 10.1038/srep45052

**Published:** 2017-03-27

**Authors:** Claudia Miersch, Katja Stange, Silvio Hering, Martin Kolisek, Torsten Viergutz, Monika Röntgen

**Affiliations:** 1 Leibniz Institute for Farm Animal Biology, Institute for Muscle Biology and Growth, Wilhelm-Stahl-Allee 2, 18196 Dummerstorf, Germany; 2 Freie Universität Berlin, Institute of Veterinary Physiology, Oertzenweg 19b, 14163 Berlin, Germany; 3 Leibniz Institute for Farm Animal Biology, Institute for Reproductive Biology, Wilhelm-Stahl-Allee 2, 18196 Dummerstorf, Germany

## Abstract

During postnatal development, hyperplastic and hypertrophic processes of skeletal muscle growth depend on the activation, proliferation, differentiation, and fusion of satellite cells (SC). Therefore, molecular and functional SC heterogeneity is an important component of muscle plasticity and will greatly affect long-term growth performance and muscle health. However, its regulation by cell intrinsic and extrinsic factors is far from clear. In particular, there is only minor information on the early postnatal period which is critical for muscle maturation and the establishment of adult SC pools. Here, we separated two SC subpopulations (P40/50, P50/70) from muscle of 4-day-old piglets. Our results characterize P40/50 as homogeneous population of committed (high expression of *Myf5*), fast-proliferating muscle progenitors. P50/70 constituted a slow-proliferating phenotype and contains high numbers of differentiated SC progeny. During culture, P50/70 is transformed to a population with lower differentiation potential that contains 40% Pax7-positive cells. A reversible state of low mitochondrial activity that results from active down-regulation of ATP-synthase is associated with the transition of some of the P50/70 cells to this more primitive fate typical for a reserve cell population. We assume that P40/50 and P50/70 subpopulations contribute unequally in the processes of myofiber growth and maintenance of the SC pool.

Skeletal muscle predominantly consists of a single differentiated cell type, the contractile myofiber. Myofibers are multinucleated and formed by fusion of many myoblasts during embryonic (primary myofibers) and foetal (secondary myofibers) development^1^,^2^,^3^. In some species a third wave of myofiber formation has been shown to occur during gestation (e.g., human^4^) or postnatally (e.g., pig^5^,^6^). However, its contribution to the total muscle fiber number is rather small^5^ and postnatal muscle growth takes place primarily by muscle cell hypertrophy^7^. As myonuclei are postmitotic, postnatal fiber growth, muscle maintenance and regeneration after injury depends on so-called satellite cells (SC)^8^,^9^, a subpopulation of myogenic stem cells localized in so-called SC niches between the sarcolemma and basal lamina. SC are responsible for generating myoblasts in postnatal muscle, and initially they are the main source of new myonuclei for growing myofibers^10^,^11^,^12^, before the vast majority become quiescent in mature muscle^13^,^14^. Nevertheless, adult SC can be recruited to supply myoblasts for routine muscle fiber homeostasis^13^, or for sporadic demands of myofiber hypertrophy or repair^15^. In addition to progeny specified for differentiation, SC also preserve their own population by self-renewal^16^,^17^,^18^.

During their quiescent phase, SC express the transcription factor paired/homeodomain gene *Pax7* and the myogenic regulatory factor *Myf5*^16^,^17^,^19^. However, about 10% of quiescent adult SC never express Myf5 (Pax7^+^/Myf5^−^ cells) and are thought to represent a population of “true” adult satellite stem cells^16^. Upon activation, when SC enter the cell cycle, MyoD expression is induced, and the MyoD protein is often used to identify activated/proliferating SC/myoblasts^7^,^18^,^20^. Myf5 and MyoD both play important roles in determining muscle cell fate, namely, a return to the quiescent state to renew the SC pool or cellular differentiation initiated by the downregulation of Pax7 and the expression of Myogenin (MyoG)^17^,^21^. Terminally differentiated cells are characterized by the expression of sarcomeric Myosin heavy chain (MyHC) proteins^2^ and fuse with each other to form nascent myotubes and with differentiated muscle fibers^22^.

In contrast to the situation in adult muscle when only 1–4% of myonuclei belong to SC, their number is much higher during the early postnatal period ranging, for example, from 30% to 35% in mouse^23^,^24^ or from 30% to 60% in pig muscle^25^. In addition, in early postnatal muscle, a high number of slowly proliferating SC is present^7^,^25^,^26^ generating fusion-competent myoblasts that contribute DNA to growing myofibers, thereby increasing their capacity for protein synthesis^12^,^27^,^28^. However, some SC can also divide asymmetrically to give rise to both myonuclei and SC^9^,^11^,^15^,^18^. In this proliferating fraction, Pax7^+^/MyoD^+^ SC are the dominating population followed by Pax7^+^/Myf5^+^ cells^7^,^29^. Lepper *et al*.^9^ have shown that the first weeks after birth are a critical period of Pax7-dependency in transition from muscle progenitor to adult stem cell state. Pax7 not only plays a critical role in early postnatal SC survival and expansion, but also directs myogenic progenitors to withdraw from myogenic differentiation and transition into quiescence^9^,^20^,^30^.

In various species such as mice, turkey, chicken, cattle, and even human^31^,^32^, both *in vitro* and *in vivo* investigations have demonstrated that SC adopt various behaviors and are generally highly heterogeneous in nature between the various muscles and within one muscle or muscle fiber^22^,^33^,^34^. This heterogeneity is reflected by the different expression of commitment markers (Pax7, Pax3, Myf5, MyoD)^17^,^31^,^33^,^35^,^36^ and by functional diversity^8^,^32^,^33^,^37^,^38^. Differences between SC have been revealed based on their adhesion properties^38^, proliferation rate^22^,^34^,^37^,^39^, fusion ability^22^,^40^, and ability to migrate and/or to respond to growth factors^41^,^42^. The signal(s) leading to intrinsic SC heterogeneity are far from clear, but changes in cellular metabolism seem to play a key role^43^,^44^. In addition, the molecular and functional properties of SC are affected by environmental factors and can be changed permanently during critical periods of muscle development^45^,^46^,^47^.

A few studies^28^,^48^ have shown that, in piglets from breeds with low and high growth performance or with low and high postnatal growth rate, the ability of SC to proliferate and differentiate is altered. However, these studies have been performed with mixed (mass) cultures of myogenic precursor cells/SC and myoblasts that do not reflect the marked heterogeneity between the various subpopulations of these cells. We hypothesize that the different growth potential is the result of permanent functional changes in distinct SC subpopulations. As a first step to test this hypothesis, we isolated and subdivided two SC subpopulations and then further characterized them with regard to proliferation, differentiation, bioenergetics, and myogenic marker expression. Thus, this study unravels SC diversification under normal growth conditions and is the basis for a better understanding of the origins and targeted modulation of growth phenotypes and of certain pathological states in the future.

## Results

**Figure 1:**
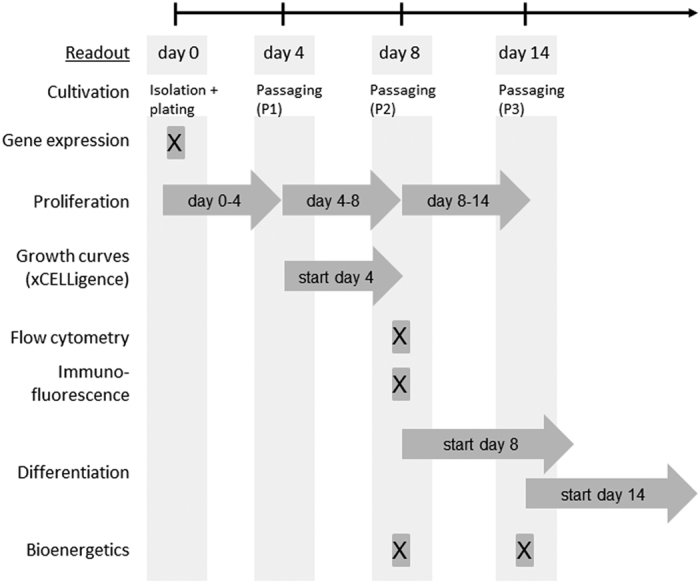
Experimental workflow. P40/50 and P50/70 cells were isolated from muscles of 4-day-old piglets. Gene expression (via qRT-PCR) was analysed in freshly isolated cells. Proliferation rate was measured when cells were passaged at day 4, 8 and 14. Cell morphology and size during proliferation, growth behaviour, protein expression (via flow cytometry and immunofluorescence), bioenergetic profile and differentiation potential were monitored at given time points.

To follow the development of SC subpopulations, the molecular and functional properties of P40/50 and P50/70 cells were characterised at various time points (see Fig. 1 for experimental workflow).

### Freshly isolated P40/50 and P50/70 cells are characterized by the distinct expression of myogenic genes

**Figure 2:**
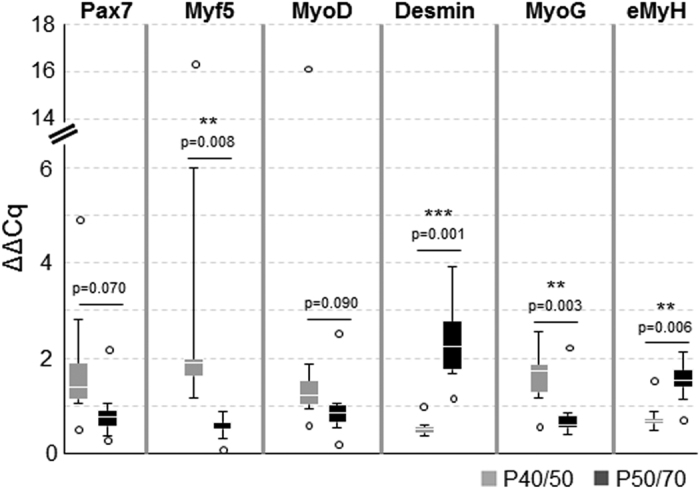
Gene expression analysis of freshly isolated P40/50 and P50/70 cells. Gene expression analysis of the myogenic marker genes *Pax7, Myf5, MyoD, Desmin, MyoG*, and *embryonic Myosin (eMyH*) of freshly isolated cells from LD muscle (n = 6). Quantitative real-time PCR shows the higher expression of transcription factors *Myf5* and *MyoG* in P40/50 cells. In contrast, *Desmin* and *eMyH* are significantly upregulated in P50/70 cells. ΔΔCT values of each sample are presented as Box-Whisker plots with the maximum 1.5 of the interquartile range (Q_1_–Q_3_), and the resulting outliers are included as circles. For statistical analysis, Students t-test (*Pax7, MyoD, Desmin, MyoG, eMyH*) or Mann-Whitney Rank Sum Test (*Myf5*) was performed, **p ≤ 0.01, ***p ≤ 0.001.

First, part of freshly isolated P40/50 and P50/70 cells was used to assess their developmental status at the time of isolation by analysing the gene expression of selected candidates crucial for myogenic development (Fig. 2). To this aim, gene expression analyses via qRT-PCR were performed to detect the transcription factors *Pax7* and *Myf5* expressed by SC and myoblasts. Furthermore, we determined the expression of *MyoD*, a characteristic marker of activated and proliferating SC, *MyoG*, and *embryonic MyH (eMyH*) identifying differentiating myoblastic cells. *Desmin* as a muscle-specific marker was also detected. Gene expression levels differed between animals, as seen in particular for *Pax7, Myf5*, and *MyoD*. There was a trend for an elevated *Pax7* (p = 0.07) and *MyoD* (p = 0.09) expression in P40/50 compared with P50/70 cells. Expression of *Myf5* (p = 0.008) and *MyoG* (p = 0.003) was significantly higher in cells of the P40/50 subpopulation, whereas *Desmin* (p = 0.001) and *eMyH* (0.006) expression was significantly elevated in the P50/70 cells.

### P40/50 and P50/70 cells show different proliferation phenotypes

Next, to characterize the proliferative capacity of P40/50 and P50/70 cells, after isolation they were seeded and cultured in growth medium for 4 days, and then passaged at day 4 (passage 1, P1) and day 8 (passage 2, P2) of culture (Fig. 1). Confluence level, cell numbers and cell size were determined at days 4, 8 and 14 of culture.

**Figure 3:**
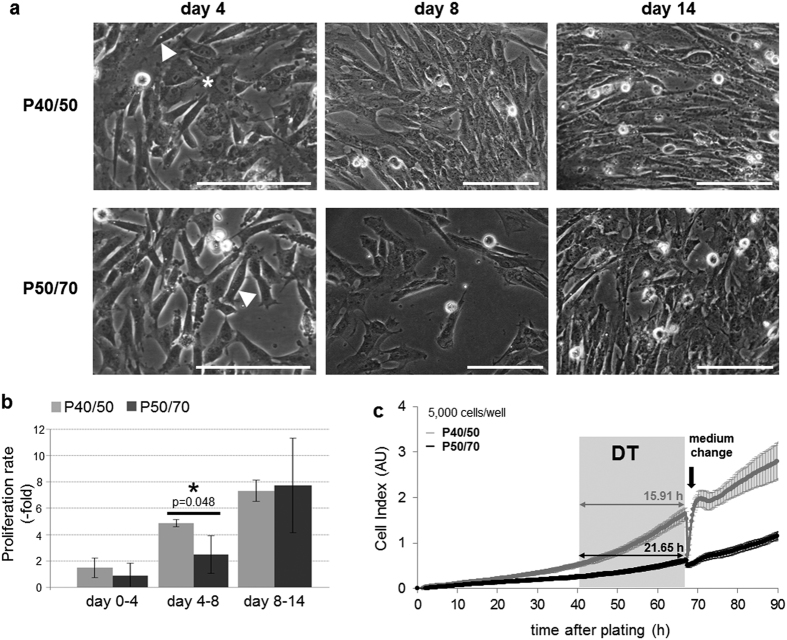
Proliferation behavior of SC subpopulations. (**a**) Light microscopy of proliferating SC isolated from SM muscles. Scale bar represents 50 μm. At day 4 of cultivation, slightly elongated, well-defined cells (◄) are found in P40/50 and P50/70 cultures. Additionally, in P40/50 cultures, slightly flattened cells can be seen (*). At day 8 of cultivation, the P40/50 subpopulation clearly shows a higher cell density reflecting an increased proliferation rate. After 14 days, morphology and density show greater similarity between the two subpopulations. (**b**) Proliferation rate of SC isolated from LD muscles was calculated by using cell numbers determined after passaging. Proliferation of P50/70 cells was considerably lower compared with that of P40/50 cells during the first 8 days of culture but achieved a comparable level after 14 days of cultivation. Bars represent mean ± SD, n = 3. For statistical analysis, Students t-test was performed, *p ≤ 0.05. (**c**) SC from LD muscles of 4-day-old piglets were isolated, and the growth kinetics of P40/50 (grey) and P50/70 (black) cells were monitored starting 4 days after isolation by using the xCELLigence system. Shown original growth curves represent mean of quadruplicates of one representative experiment ± SD, results were reproducible. Proliferation assay (5,000 cells/well) was performed and the medium was exchanged at given time point (←). The doubling time (DT) obtained during the logarithmic growth phase is presented. Cells of the P40/50 population exhibit superior growth kinetics showing faster proliferation.

Compared with cells of P50/70, P40/50 cells reached higher confluence levels at days 4 and 8 of culture (Fig. 3a) and showed a higher proliferation rate (Fig. 3b and c). In addition, at day 4, the cell size of P40/50 cells was greater than that of P50/70 cells (12.2 ± 0.2 μm vs. 11.7 ± 0.1 μm, p = 0.046), indicative of a higher proportion of P40/50 cells being in the S-phase of the cell cycle. Functional differences were prominent between day 4 and 8 of culturing when the cell number increased significantly more (5.24 ± 0.58-fold vs. 2.54 ± 1.50-fold, p = 0.048) in P40/50 cells compared with P50/70 cells (Fig. 3b). This is further illustrated by representative original growth curves of P40/50 and P50/70 cells obtained by using the xCELLigence system (Fig. 3c). Starting at day 4 of culturing growth curves were recorded over a period of 90 h and show that P40/50 cells were characterized by a fast-proliferation phenotype, whereas in P50/70 cells, this process proceeded to a slower extent. The latter is in agreement with a considerably higher doubling time in P50/70 compared with P40/50 cells (Fig. 3c).

Representative micrographs (Fig. 3a) show elongated well-defined cells in both subpopulations, whereas the P40/50 culture contained more flattened cells. Cell morphology and density exhibited greater similarity between the two subpopulations from day 8 on (Fig. 3a). In addition, the mean proliferation rate was similar in P40/50 and P50/70 cells after P2 (day 8–14) but showed a higher variability in P50/70 cells.

### P40/50 and P50/70 differ in myogenic marker expression after prolonged cultivation

**Figure 4:**
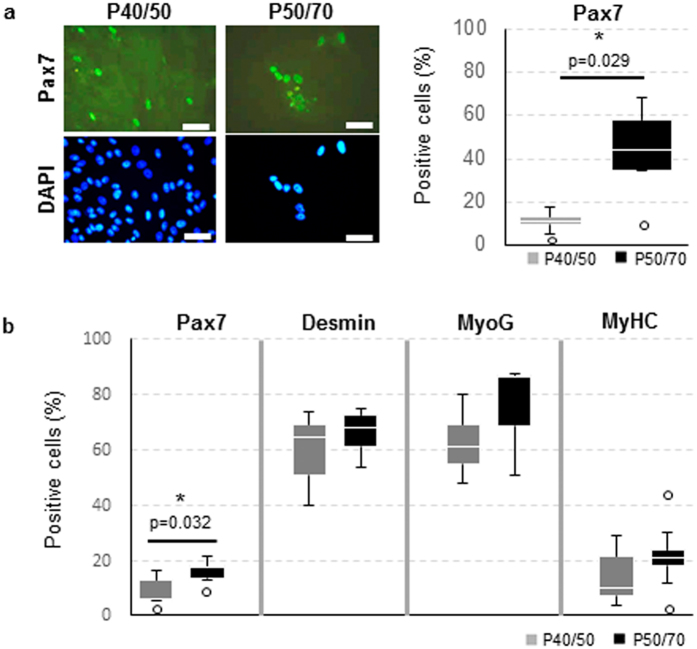
P40/50 and P50/70 differ in myogenic marker expression after prolonged cultivation. (**a**) P40/50 and P50/70 cells were passaged at day 4 and cultured in growth medium for another 5 days. Subsequently, immunofluorescence staining was performed to visualize Pax7; cell nuclei were stained with DAPI. Pax7 was localized in the nuclei, and a higher proportion of nuclei was positive for the transcription factor in P50/70 cells, in contrast to P40/50 cells. Percentages of positive cells of each sample (n = 5) are presented as Box-Whisker plots with the maximum 1.5 of the interquartile range (Q_1_–Q_3_), and the resulting outliers are included as circles. For statistical analysis, Mann-Whitney Rank Sum Test was performed, *p ≤ 0.05. (**b**) Flow cytometric analysis of proliferating SC from SM or LD muscle after 8 days of cultivation. P40/50 cells show a significantly higher proportion of cells positive for Pax7. Percentages of positive cells of each sample are presented as Box-Whisker plots with the maximum 1.5 of the interquartile range (Q_1_–Q_3_), and the resulting outliers are included as circles. For statistical analysis, Students t-test (Desmin, MyoG, MyHC) or Mann-Whitney Rank Sum Test (Pax7) was performed, *p ≤ 0.05, n = 4 (MyoG, MyHC), 5 (Pax7) and 9 (Desmin).

Since identified SC populations showed most obvious differences regarding proliferation around day 8 after isolation, this time point was chosen for investigation of myogenic marker protein expression. During perinatal life Pax7 is essential for SC maintenance^9^,^35^. Although freshly isolated P50/70 cells expressed mRNA of various differentiation markers, immunofluorescence staining showed that 48 ± 17% of P50/70 cells expressed protein of the SC marker Pax7 after 8 to 9 days in culture, whereas only 11 ± 5% of P40/50 cells were found to be Pax7^+^ (Fig. 4a). Furthermore, proliferating cells in P40/50 and P50/70 were analysed with regard to the protein expression of the myogenic markers Pax7, Desmin, MyoG and Myosin HC at day 8 of culture in growth medium by using flow cytometry (Fig. 4b). Significantly (p = 0.032) higher expression of Pax7 in P50/70 cells could be confirmed. In both subpopulations a large proportion of cells expressed the myogenic marker Desmin (64 ± 10%) and the early differentiation marker MyoG (68 ± 17%). As expected, only a low proportion (17 ± 10%) of cells was positive for Myosin HC protein after 8 days of culture in growth medium.

### P40/50 and P50/70 cells show dissimilar differentiation potential

In order to form functional myogenic tissue, progenitor cells have to differentiate into myoblasts and to form multinucleated myotubes/fibers. Since cells of the P40/50 and the P50/70 subpopulations showed distinct growth performances, the question arose as to whether they also varied in their ability to differentiate.

**Figure 5:**
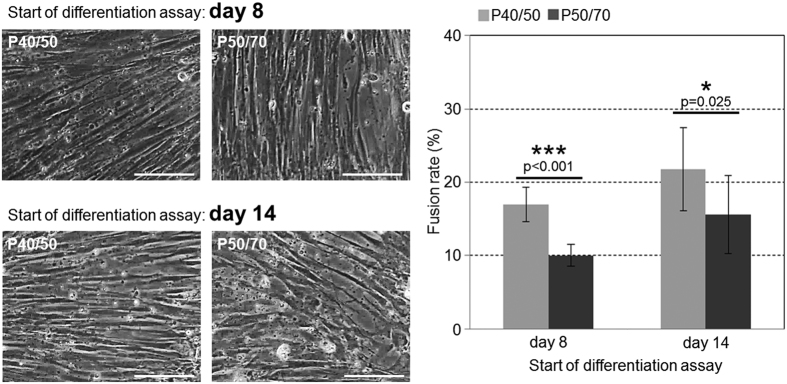
SC in P40/50 population preferentially differentiates compared with P50/70 cells. Light microscopy of differentiating SC isolated from SM muscle (left). Cells were seeded at 8 or 14 days after isolation in growth medium. Before reaching confluency, cells from both subpopulations were transferred to differentiation medium to allow differentiation. P40/50 cells show a more elongated and dense morphology than P50/70 cells (see especially in the lower panel). Scale bar represents 50 μm. Fusion rate (right) of cells isolated from SM and LD muscle was determined during early differentiation (7 days after the start of the differentiation assay) as the percentage of total nuclei being localized in fused multinucleated cells (day 8: n = 8, day 14: n = 9). P40/50 cells show significantly higher cell fusion compared with P50/70 cells. For statistical analysis, Students two-tailed t-test was performed, *p ≤ 0.05, ***p ≤ 0.001.

To test the differentiation capacity of the two subpopulations, differentiation assays were performed starting at day 8 or day 14 after isolation (Fig. 5). At both time points cells from the P40/50 and P50/70 subpopulation developed elongated multinucleated structures and showed the ability to differentiate into the myogenic lineage (Fig. 5). Both subpopulations showed higher fusion rates in cultures starting at day 14 in comparison to day 8. However, the quantification of nuclei in fused cells demonstrated a significant higher fusion rate in the P40/50 subpopulation at both time points (17% and 22%) in comparison to P50/70 cells (10% and 16%, p < 0.001 and p = 0.025).

### Bioenergetic profile of P40/50 and P50/70 SC subpopulations

Given the substantial molecular and functional heterogeneity of the subpopulations that we had found and the initial research showing the importance of cellular metabolism in the regulation of skeletal muscle stem cells^18^,^49^, we investigated the bioenergetic profile of P40/50 and P50/70 cells at day 8 and day 14 of culture.

**Figure 6:**
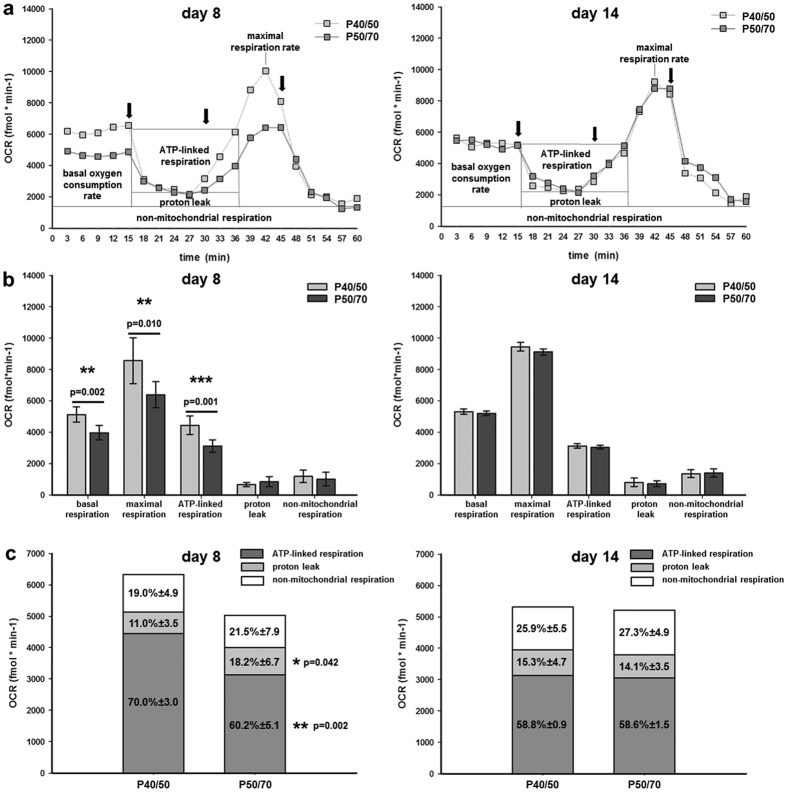
Bioenergetic parameters in P40/50 and P50/70 SC subpopulations. The oxygen consumption rate (OCR) of SC was first measured under basal conditions and then after application of inhibitors of mitochondrial function (Oligomycin, FCCP, Antimycin A1) at time points indicated (←). Characteristic original curves (**a**), values of basal OCR, maximal FCCP-induced respiration, ATP-linked respiration, proton leak, and non-mitochondrial respiration (**b**) and the proportion of ATP-linked respiration, proton leak, and non-mitochondrial respiration on basal OCR (**c**) are given for P40/50 and P50/70 cells after 8 days (left) and 14 days (right) of culture, respectively. Bars represent mean ± SD for n = 6 (day 8) and n = 3 (day 14) single measurements. For statistical analysis, Students t-test was performed, *p ≤ 0.05, **p ≤ 0.01; ***p ≤ 0.001.

At day 8 of culture, the bioenergetic profile of the P40/50 and P50/70 cells differed markedly (Fig. 6a). The rates of basal endogenous OCR and of FCCP-induced maximal respiration were approximately 30% and 36% higher in P40/50 cells than in P50/70 cells (5,134 ± 477 fmol/min and 8,566 ± 1,462 fmol/min in P40/50 cells versus 3,977 ± 462 fmol/min and 6,400 ± 836 fmol/min in P50/70 cells). We also noted a 44% higher oligomycin-sensitive respiration rate in P40/50 cells (4,451 ± 588 fmol/min) compared with P50/70 cells (3,130 ± 394 fmol/min). This reflected a 30% decrease of ATP-synthase-linked respiration in P50/70 cells. Proton leak was reduced by approximately 13% in P40/50 compared with P50/70 cells (684 ± 137 fmol/min in 40/50 cells versus 868 ± 319 fmol/min in P50/70 cells) and showed a high variability in the latter. Non-mitochondrial respiration was calculated to amount to 1,200 ± 398 fmol/min in P40/50 cells and to 1,029 ± 430 fmol/min in P50/70 cells. None of these differences was observable at day 14 of culture (Fig. 6b).

Figure 6c shows the proportion of ATP-synthase-linked respiration, proton leak, and non-mitochondrial respiration in relation to basal respiration for the P40/50 and P50/70 cells. At day 8, they did not differ in non-mitochondrial respiration (≈20%), but P50/70 cells had a significantly increased proportion of proton leak (18% vs. 11% in 40/50 cells). ATP-synthase-linked respiration caused 70% of basal respiration in P40/50 cells but only 60% in P50/70 cells. No differences were observed at day 14, and the proportion of non-mitochondrial respiration, proton leak, and ATP-synthase-linked respiration amounted to approximately 26%, 15%, and 58%, respectively.

The respiratory control ratio was similar for both P40/50 and P50/70 cells amounting to 0.59 ± 0.06 and 0.57 ± 0.08 at day 8 and to 0.44 ± 0.03 and 0.43 ± 0.01 at day 14, respectively.

To test if observed differences in cellular metabolic activity of P40/50 and P50/70 cells are related to changes in mitochondrial mass, we determined the protein concentration of isolated mitochondria. In addition, the electrochemical proton gradient (Δψ_m_) of the inner mitochondrial membrane, the driving force for ATP-synthesis and an indicator of mitochondrial functional integrity was measured. The results are summarized in Table 1, showing similar mitochondria mass in both subpopulations. However, the inner membrane potential and thus, the metabolic activity of the mitochondria were lowered by 43% in P50/70 compared with P40/50 cells (Tab. 1).

### Proportion of P40/50 and P50/70 subpopulations changes over time *in vivo*

**Figure 7:**
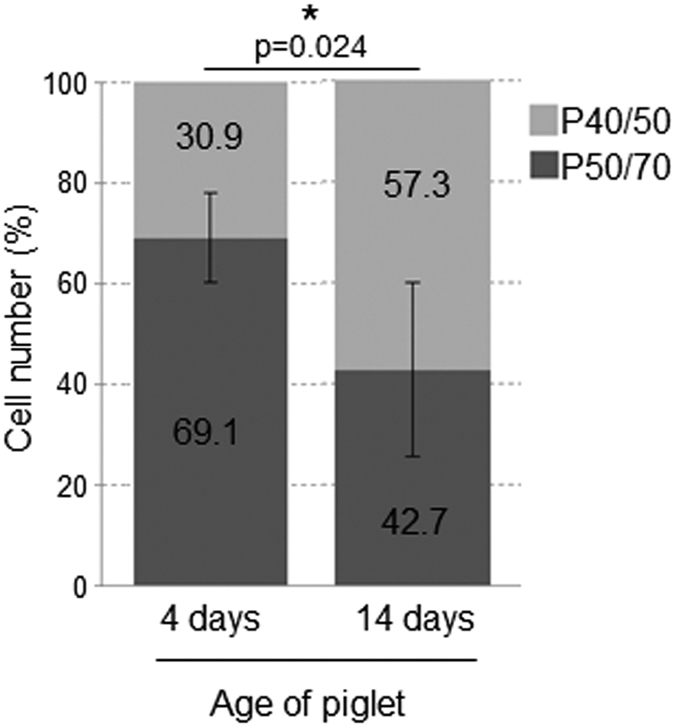
Distribution of SC populations dynamically changes *in vivo*. SC were isolated from LD muscles of 4-day-old (n = 7) or 14-day-old piglets (n = 4). In the younger animals, P50/70 cells represent the dominant subpopulation, whereas in older piglets, cells from P40/50 are preferentially found. The relation of both subpopulations is significantly different in younger and older piglets. Bars represent mean ± SD. For statistical analysis, Mann-Whitney Rank Sum Test was performed, *p ≤ 0.05.

To obtain information regarding the bioavailability of P40/50 and P50/70 populations, we investigated SC yield and the proportion of these subpopulations at day 4 and day 14 of age. In 4-day-old piglets, only 31% of freshly isolated cells were found in the P40/50 fraction, whereas 69% were present in the P50/70 subpopulation (Fig. 7). The overall cell yield decreased from 6.24 ± 1.49*10^5^ cells/g muscle at day 4 to 3.93 ± 0.6*10^5^ cells/g muscle at day 14. Moreover, during skeletal muscle development the proportion of both subpopulations changed in favour of P40/50 (Fig. 7). At day 14 of age, this population represented 57% of the isolated cells, whereas 43% were found in the P50/70 population.

## Discussion

**Figure 8:**
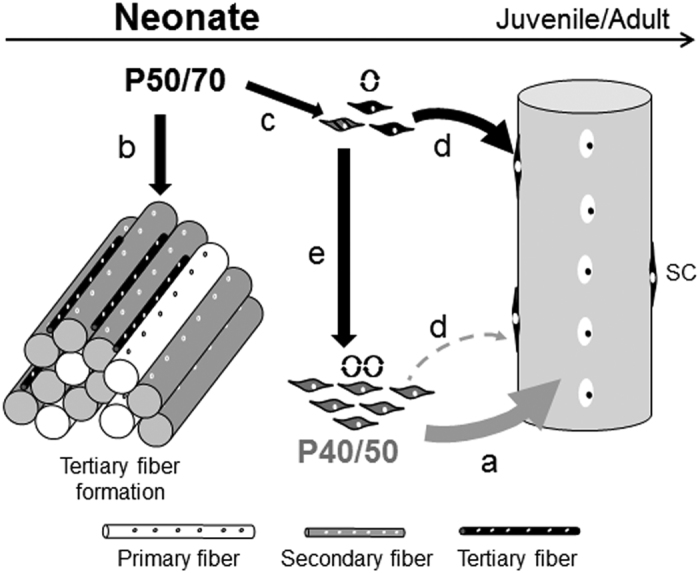
Summary of proposed roles of characterized SC subpopulations in myogenesis. In this study, two distinct SC subpopulations (P40/50 and P50/70) were isolated from muscle of 4-day-old piglets. Freshly isolated cells of the P40/50 subpopulation highly expressed *Myf5* and *MyoG* and constituted a fast-proliferating phenotype. During *in vitro* cultivation, the P40/50 cells show a constantly high oxidative capacity, an increased differentiation potential, and fusion rates higher than the P50/70 cells. From these results, we assume that these cells by being a source of new myonuclei are the main contributors to hypertrophic growth of existing myofibers (**a**). Freshly isolated P50/70 cells showed considerably slower proliferation and expressed high amounts of markers for terminal differentiation (*Desmin, eMyH*). This leads us to suppose that some of the P50/70 cells are involved in tertiary fiber formation occurring with the highest intensity during the first week of pig postnatal muscle development (**b**). During culture, at least some of the P50/70 cells pass through a reversible period of low mitochondrial activity; this is associated with the appearance of higher numbers of Pax7^+^ cells and reduction of the differentiation potential/fusion rate compared with P40/50 cells whereas the proliferation rate equalized. Based on these results, we hypothesize that a subpopulation of P50/70 is withdrawn from differentiation to form and maintain a pool of slowly cycling, more immature precursor/reserve cells (**c**) at existing muscle fibers (**d**) gradual transformation to quiescent adult SC) that also can give rise to fast-proliferating, more committed (P40/50) cells (**e**) to prevent their exhaustion during intensive growth.

In the juvenile pig, the SC pool has been shown to be a critical component of both hyperplastic and hypertrophic processes^6^,^11^,^28^. However, established mixed (mass) cultures of pig SC and myoblasts do not reflect the marked functional heterogeneity that has been shown to exist in SC pools of other species^37^,^50^,^51^. To our best knowledge, the concept of heterogeneous SC populations has not been explored in the pig, and generally, few studies have involved functional investigations of SC in the context of increased skeletal muscle turnover, such as early postnatal growth. In this study, by use of a 25%, 40%, 50%, 70% percoll gradient, we have isolated and enriched two subpopulations of SC from 4-day-old piglets when muscles are characterized by a high absolute number of SC being mostly in the cycling state (≈90%)^25^. Indeed, freshly isolated cells of both subpopulations expressed transcripts of the SC marker *Pax7* and the myogenic determination factors *MyoD* and *Myf5* characteristic for proliferating SC fractions^7^,^29^. Importantly, P40/50 and P50/70 cells maintained their myogenic identity *in vitro*. When cultivated for 8 days under growth-promoting conditions, a majority of them expressed Desmin and MyoG, and both subpopulations underwent myogenic differentiation when induced by the reduction of the medium serum concentration at day 8 or 14 of culture. Nevertheless, the P40/50 and P50/70 subpopulations clearly show distinct gene and protein expression profiles and differ in their proliferation behaviour, growth kinetics, bioenergetics, and myogenic differentiation. Thus, the question arises as to whether the characteristic phenotypes of P40/50 and P50/70 cells are linked to specific myogenic processes. The current working model deduced from the present results has been summarised in Fig. 8.

Our data characterize P40/50 as a relatively homogeneous population of committed myogenic progenitors and cells marked for terminal differentiation (high expression of *Myf5* and *MyoG*)^11^,^39^ constituting a fast-proliferating phenotype. After some rounds of proliferation, P40/50 exhibited lower numbers of Pax7^+^ cells but higher fusion rates compared with P50/70 cells. In a study by Rouger *et al*.^22^, fast-proliferative SCs also had a higher fusion rate and were exclusively committed to fuse with differentiated myotubes to form myotubes/myofibers with a large diameter. In a similar manner, a fast-dividing SC population obtained from young rat muscles readily fused into the adjacent myofiber^51^. We therefore speculate that P40/50 cells due to their higher differentiation potential and fusion ability are the main source of myonuclei accretion to existing muscle fibers during growth (Fig. 8a) which is in accordance with the increase of P40/50 cells observed *in vivo*.

Cells found at the 50/70% percoll interface seem to be more heterogeneous. After isolation higher expression levels of genes (*Desmin, embryonic MyH*) encoding structural proteins are found suggesting that an increased proportion of P50/70 cells are further along the differentiation pathway than P40/50 cells at day 4 of age. This provides a good explanation for the slow-proliferation phenotype and reduced number of progeny observed in early (up to day 8) P50/70 cultures. Interestingly, the proportion of P50/70 cells is reduced from 69% at day 4 to 43% at day 14 of age. The results led us to hypothesize that *in vivo* a fraction of the P50/70 cells might be involved in the formation of a third generation of myotubes and/or the elongation of existing myofibers (Fig. 8b), both of which occur with decreasing intensity during the first two weeks of life^5^. The source of pig tertiary fibers has not as yet been clarified, but a role of SC has been discussed^6^. This idea is supported by results from young 7-day-old turkeys^22^ showing that slow-proliferating SC mainly fuse together to form nascent fibers.

However, a high proportion (40%) of Pax7^+^ cells was established in P50/70 at day 8 of culture, and proliferation potentials were no longer different from P40/50 after two weeks (day 14). Moreover, when differentiation assays were started at day 8 or 14 of culture, P50/70 cells exhibited significantly reduced fusion rates compared with P40/50 cells. In P50/70 cells, the process of changed functionality was associated with reversible alterations in mitochondrial bioenergetics. Both P50/70 and P40/50 mainly (≈80%) used oxidative phosphorylation (OXPHOS) to generate the energy (ATP) needed, which promotes the cycling type of SC cells needed for early postnatal muscle development^34^,^52^. However, during a limited time period around day 8 of culture, both the load-depending basal oxygen consumption rate (22%) and the maximal respiration (23%) were lower in P50/70 cells. The maximal respiration is a measure of the total oxidative capacity of the cell and represents an intrinsic property of the mitochondria. Indeed, the inner mitochondrial membrane potential (proton motif force; Δψ_m_) that drives ATP synthesis is lower in mitochondria from P50/70 cells compared with P40/50 cells. In accordance, a higher proportion of proton leak was observed in P50/70 cells reflecting mild uncoupling. Most importantly, in P50/70 cells the ATP-linked respiration is significantly reduced by 30% whereas the mitochondrial mass was similar in P40/50 and P50/70 cells. Since we have found no indication for a loss of respiratory control (both subpopulations operated at an average of 58% of maximal respiration), this inhibition of ATP-synthase activity in P50/70 cells presumably results from the active downregulation of its functionality.

In various stem cells, a reversible state of low mitochondrial activity has been shown to constitute internal metabolic signals affecting gene expression and functionality, in particular, with regards to differentiation and cell fate^35^,^43^. Here, we show that the P50/70 population contains a higher proportion of Pax7^+^ cells compared with the P40/50 population and suggest that the reversible reduction of mitochondrial metabolism is required to form a SC subpopulation that maintains the more primitive fate typical for a reserve cell population (Fig. 8c). In mice, Chakkalakal *et al*.^50^ have also found that slow-dividing cells maintain a more primitive type during proliferation and retain more Pax7^+^ cells than fast-dividing SC only at later stages of early postnatal muscle maturation.

Little is known about the metabolic states of SC and the way that they may change during SC life cycle and postnatal development. In one of the few studies with SC, Rocheteau *et al*.^35^ have identified small SC subpopulations characterized by low or high Pax7 expression in juvenile and adult mouse muscle. Although we did not sort our cells for Pax7 expression the behaviour of these subpopulations resemble that of our P40/50 and P50/70 cells and, similar to the latter, Pax7-high cells had reduced levels of active mitochondria. During the early postnatal period, Pax7 is known to be essential for the extended proliferation potential, expansion, and survival of muscle progenitors and to preserve their myogenic potential^9^,^20^. Moreover, during a critical period of postnatal muscle development, Pax7 is required for the transition from muscle progenitor to adult SC status, which is induced by the withdrawal from differentiation and the transition into quiescence^9^,^17^. Lepper *et al*.^9^ have shown that a higher expression of *Pax7* directs cells to have a lower propensity to differentiate, a result that agrees with the delayed myotube formation observed in Pax7-high cells^35^ and with our finding of a decreased fusion rate in P50/70 cells. Whereas the differentiation potential remains reduced in P50/70 cells, the proliferation rate and the bioenergetic profile of P50/70 and P40/50 subpopulations equalize during longer *in vitro* cultivation (14 days). Therefore, we assume that during *in vivo* myogenesis cells of the P50/70 subpopulation contribute markedly to the elevation of the absolute number of SC (Fig. 8d), and give rise to more committed (P40/50) cells to provide a constant supply of myoblasts for postnatal muscle growth (Fig. 8e).

Our work reveals notable heterogeneity in P50/70 characterized by a dominance of terminally differentiated myoblasts at isolation and appearance of high numbers (40%) of more immature Pax7^+^ cells with a lower propensity to differentiate during cultivation. An additional 60% percoll layer, immunofluorescence and flow cytometry analyses after co-staining with multiple markers followed by FACS will be undertaken to separate and investigate these subpopulations in more detail.

In accord with our results, two subsets of SC with different proliferation potential or cell cycle kinetics have also been found in other species investigated, e.g., in mice^37^,^50^, chicken^53^, cattle^33^, and human^34^, and shown to differ in their Pax7 expression, fusion ability, and self-renewing potential^22^,^35^,^37^,^40^,^50^. In addition, fast and slow proliferating SC exist after the activation of adult SC and within regenerating and growing muscles^14^,^22^,^37^,^50^, and are proposed to reflect at least two distinct SC populations: committed progenitors responsible for muscle growth and routine maintenance, and reserve SC/SC stem cells^7^,^16^,^40^,^51^. As in our study, the latter have been found to be part(s) of the slow proliferating SC pool^35^,^37^. Our aim in further investigations will be to uncover the main regulatory pathways underlying the diversity of P40/50 and P50/70 cells, and to understand their precise role in postnatal development of growing muscle. This is a prerequisite for resolving the root causes of the different postnatal growth potentials observed, e.g., in piglets having low or normal birth weights^54^,^55^, and will open up new possibilities to modulate muscle growth and regeneration by the targeted stimulation of mechanisms intrinsic to specific SC subpopulations. Any relevant findings will also have implications for human muscle diseases and regenerative therapy.

## Materials and Methods

### Animals

For the experiments, 4-day-old or 14-day-old German Landrace piglets were obtained from the experimental pig unit of the Leibniz Institute of Farm Animal Biology (FBN). Animal husbandry and slaughter followed the guidelines set by the Animal Care Committee of the State Mecklenburg-Western Pomerania, Germany, based on the German Law of Animal Protection.

### Isolation and cultivation of SC

The right and left *musculus semimembranosus* (SM) and the right and left *musculus longissimus dorsi* (LD) were removed as a whole, trimmed of visible connective tissue, and weighed in phosphate-buffered saline (PBS) containing glucose (25 mM), sucrose (14 mM), penicillin (1000 U/ml), streptomycin (1 mg/ml), and amphotericin (25 μg/ml).

The protocol established by Mau and colleagues^56^ was modified during this study. Dissected tissue was trimmed intensively before digestion with trypsin 1x solution (4000 U/ml, Sigma Aldrich). After being washed and filtered, SC were enriched by using layers of 70%, 50%, 40%, and 25% percoll (Sigma Aldrich) in PBS during gradient centrifugation. The molecular and functional properties of SC from the 50/70% percoll gradient interface (P50/70 cells) and the 40/50% percoll gradient interface (P40/50 cells) were investigated (experimental approach is shown in Fig. 1). Enriched cells were re-suspended in growth medium (αMEM Eagle, fetal bovine serum [20%, FBS, PAN Biotech], penicillin/streptomycin [100 U/ml], amphotericin [2.5 μg/ml], and gentamycin [0.05 mg/ml]). The cell number was quantified by using the Countess Automated Cell Counter (Thermo Fisher Scientific) following the manufacturer’s instructions. This system uses trypan blue staining combined with an image analysis algorithm to obtain accurate cell counts together with cell vitality. Cells were seeded in dishes coated with Collagen type I (Greiner Bio-one) and cultured under a humidified atmosphere with 5% CO_2_ at 37 °C. 24 hours after seeding, the cells were washed with Dulbecco’s phosphate-buffered saline (DPBS) containing penicillin/streptomycin (100 U/ml), amphotericin (2.5 μg/ml), and gentamycin (0.05 mg/ml). Bacterial and fungal contamination of cells was excluded via inoculation of CASO Bouillon Tryptic Soy Broth and Thioglycolate medium EP.

For passaging, cultured SC were detached by using HyClone HyQTase, and the reaction was stopped by adding DPBS containing 10% FBS. After centrifugation (10 min, 300 g, 10 °C), cells were re-suspended in growth medium, and the cell number was determined. From the first passage on, SC were cultured on Primaria-treated tissue culture dishes (VWR International) in growth medium. For light microscopy, Leica DM4000B and the associated software Leica QWin V3 were used.

### Differentiation of SC

For differentiation, 1*10^5^ cells/well were seeded in growth medium on a Primaria-treated 24-well plate (VWR International). At 80–90% confluency, FBS was reduced to 2% in differentiation medium. Medium was changed regularly. A Leica DM4000B and the associated software Leica QWin V3 were used to examine cells light-microscopically. To determine cell fusion rate, nuclei were stained with 4,6-diamidino-2-phenylindole (DAPI), and the number of nuclei in fused multinucleated cells was divided by the total number of visible nuclei in at least 4 random sections for each subpopulation. Elongated cells containing more than one nucleus were termed “fused cells”. On average, more than 1200 nuclei were counted for each sample.

### Immunofluorescence

Isolated SC were seeded on cover slides (ᴓ12 mm, Menzel) in a Primaria-treated 24-well plate (VWR International) in which wells were coated with Collagen A (0.5 mg/ml, Biochrom). After cultivation in growth medium, cells were fixed with formaldehyde (3.7%) in PBS. Cells were permeabilised in PBS containing Triton X-100 (0.5%) for 20 min. Cells were then blocked in PBS with 20% normal serum and 0.5% Triton X-100 for 1 h. Incubation with primary antibody was performed overnight. Mouse anti-Pax7 antibody (Developmental Studies Hybridoma Bank) was diluted 1:50 in normal serum (2%) and TritionX-100 (0.5%) in PBS. After being washed with PBS, samples were incubated with a rabbit anti-mouse Alexa488 antibody (Thermo Fisher Scientific, 1:1000) for 1 h at room temperature, and subsequently, cell nuclei were stained with DAPI. For fluorescence microscopy, the Leica DM4000B and its associated software Leica QWin V3 were used. Micrographs were merged by using Adobe Photoshop CS5; contrast and brightness were adjusted to the same degree in every sample group.

### Impedance-based measurement of growth kinetics

The xCELLigence system (RTCA-SP, ACEA Biosciences Inc.) was used according to the manufacturer’s instructions for the continuous real-time monitoring of cell adhesion and proliferation by cell-electrode impedance^57^ displayed as the dimensionless Cell Index (CI). By using an RTCA Analyzer, electrical impedance changes were measured across interdigitated microelectrodes integrated on the bottom of a specialized 96-well plate (E-Plate 96) and sent to the RTCA control subunit. The latter used RTCA Software (version 2.0) for CI calculations from the frequency-dependent electrode resistances and real-time display of data.

The background impedance of E-Plate 96 wells was determined growth medium only. Subsequently, 5,000 cells/well were plated in growth medium. A relatively low cell number was used to enable exponential growth and examination of the proliferation capacity. Then, localized on the RTCA SP Station, the E-Plate 96 was placed into the CO_2_-incubator, and the CI was monitored every 15 minutes over a period of 90 hours. RTCA Software was used to calculate the doubling time (DT).

### Gene expression analysis

Myogenic progenitor cells were isolated from 4-day-old piglets (n = 6) according to the procedure developed during this study. Approximately 5–6*10^6^ cells derived from the 40/50% or 50/70% percoll interface were collected in RNAprotect Cell Reagent (Qiagen), and total RNA was isolated with the NucleoSpin RNA II kit (Macherey-Nagel) according to the manufacturer’s protocol. The integrity, purity, and amount of RNA were determined by using an Agilent 2100 bioanalyzer (Agilent Technologies) and a Nanophotometer P-Class (Implen). The RNA integrity number (RIN) of all samples exceeded 9.2. Possible genomic DNA contamination of each sample was tested by the polymerase chain reaction (PCR) when using the RNA eluate instead of cDNA as a template. Synthesis of cDNA was performed with an iScript cDNA-synthesis kit (Bio-Rad) according to the manufacturer’s protocol.

Primers for the amplification of the myogenic marker genes *sus scrofa (sc) Pax7*^27^, *scMyf5*^27^, *scMyoD*^27^, *scMyoG*^58^, *scDesmin*^59^, and *sc embryonic Myosin (eMyH*)^60^ were used. Appropriate primer-annealing conditions for real-time quantitative PCR (qRT-PCR) were assessed for each respective primer pair by a temperature gradient PCR (tg-PCR) in a MasterCycler nexus gradient (Eppendorf) and an agarose electroseparation of respective amplicons. Optimal primer-annealing conditions and specificity were estimated by agarose electroseparation.

SYBR-green-based qRT-PCR was performed in an iCycler iQ (Bio-Rad) with the following protocol: step 1) initial denaturation 30 sec, 95 °C and polymerase activation 3 min, 95 °C; step 2) 2-step cycling (denaturation 30 sec, 95 °C; annealing and elongation 2 min, 58 °C; repeated 37x) with intermediate steps: 30 sec, 95 °C and 30 sec, 55 °C; step 3) melting curve 10 min, 55 °C–94.5 °C. The geNorm (qbase + , biogazelle) analysis revealed *TOP2B*^61^ and *YWHAZ* (Primer: sense 5′-atgcaaccaacacatcctatc-3, antisense 5′-gcattattagcgtgctgtctt-3′) to be the most suitable reference genes for our assay. ΔΔCq values (relative quantitation) were calculated according to the following formula: ΔΔCq = √(Control-Sample)^CqGOI^/√(Control-Sample)^CqRefG^; Cq (quantification cycle), GOI (gene of interest), RefG (reference gene). For each of the animals, the sample of cells from the 50/70% percoll gradient interface (P50/70 cells) was controlled with the corresponding sample of cells from the 40/50% percoll gradient interface (P40/50 cells) and *vice versa*.

### Flow cytometry analysis

Cultured SC were detached by using HyClone HyQtase, and the reaction was stopped by adding DPBS containing 10% FBS. After centrifugation (10 min, 300 g, 10 °C), cells were re-suspended in EDTA (1 mM) in DPBS and fixed in 4% paraformaldehyde (MyoG and Pax7 staining) or ice-cold methanol (Desmin or Myosin staining). After fixation with paraformaldehyde cells were also permeabilised with 0.1% (MyoG) and 0.5% (Pax7) TritonX-100 in PBS and blocked with 20% normal serum. Incubation with primary antibody mouse anti-Pax7 (Developmental Studies Hybridoma Bank, 1:50), mouse anti-Myogenin (abcam, 1:50), mouse anti-Desmin (DAKO, 1:80) or mouse anti-Myosin (skeletal, fast; Sigma Aldrich, 1:400) was performed overnight. A portion of each sample was incubated with normal serum instead of primary antibody as a negative control. After two additional washing steps, samples were incubated with a corresponding Alexa488 antibody (Thermo Fisher Scientific, 1:1000) for 1 h at room temperature and washed again. In all, 10,000 events per sample were analyzed by using an argon-equipped Gallios Flow Cytometer (Beckman Coulter) and evaluated with Kaluza software (Beckman Coulter).

### Determination of oxygen consumption rate (OCR) and mitochondrial bioenergetics

The OCR of SC was measured by using commercially available fluorophore-coated 96-well polystyrene plates (OxoPlates, Precision Sensing GmbH), which allowed prolonged cell cultivation. The basis for the technology is a thin polymer film, which contains two different dyes: the indicator (platinum porphine) and the reference (sulforhodamine) dye^62^. As the oxygen-sensitive indicator dye is quenched by molecular oxygen, the emitted fluorescence varies inversely with oxygen concentration, and the fluorescence intensity (I_ind_) increases when cells survive or grow. The fluorescence intensity of the reference dye (I_ref_) is independent of the oxygen content.

SC suspended in measuring medium (MM) at a concentration of 25,000 cells/well were seeded on OxoPlates (final volume of 180 μl/well) and incubated for 6 hours in an atmosphere of humidified air-6% CO_2_ at 37 °C until the measurements were started. Beforehand, cell numbers between 5,000 to 60,000 were tested to identify optimal assay conditions (data not shown). Each cell preparation was seeded in quadruplicate per treatment. MM is a custom-made DMEM (PAN Biotech) having the following modifications: without NaHCO_3_ and phenol red but containing glucose (5.5 mM), Na-pyruvate (1 mM), stable glutamine (4 mM), HEPES (5 mM), pH 7.3, osmolarity 290 mosmol/l. Wells containing oxygen-free water (Cal0) and air-saturated water (Cal100) were used for fluorescence reader-specific sensor calibration. For Cal100, about 10 ml of deionized water was placed into a 50 ml falcon tube, which was closed with a screw cap and shaken vigorously for approximately 2 min. The falcon tube was then opened and moved gently to avoid oversaturation. Cal0 was prepared by dissolving 0.1 g sodium sulfite in 10 ml DPBS.

After the 6-hour pre-incubation, calibration solutions (Cal0, Cal100) and MM without cells (negative control) were added to scheduled wells, and all wells were sealed with mineral oil (Sigma-Aldrich). After a 1-hour equilibration period, the fluorescence data were measured for 15 min to establish the basal OCR. Subsequently, the inhibitors oligomycin, FCCP (carbonyl cyanide-p-trifluoromethoxyphenyl-hydrazone), and antimycin A1 were used to determine the bioenergetic profile of the cells. Oligomycin (10 μM) was added 15 min after starting the experiment; it inhibits ATP synthesis by blocking the proton channel of the F_o_ portion of the ATP-synthase (Complex V) and is used to distinguish the percentage of oxygen consumption devoted to ATP synthesis and the percentage of oxygen needed to overcome the natural proton leak across the mitochondrial membrane. FCCP (10 μM) was injected 30 min after starting the experiment; this ionophore uncouples oxidative phosphorylation by inducing a proton conductance across the inner mitochondrial membrane, thereby disrupting the electrochemical gradient (proton motif force; Δψm) that drives ATP synthesis, and is used to calculate the maximal respiratory rate. After 45 min, antimycin A1 (10 μM), a complex III inhibitor, was added to the cells. Antimycin A1 inhibits the flow of electrons from cytochrome b to cytochrome c1. Hence, mitochondrial respiration will shut down and enable the differentiation of mitochondrial and non-mitochondrial fractions contributing to respiration.

The fluorescence emitted from the indicator (I_ind_) and reference (I_ref_) dyes was measured by use of a heated (38 °C) multimode reader (VICTOR^3^, PerkinElmer) at excitation and emission wavelengths of 540/650 nm or 540/590 nm, respectively.

Fluorescence values were converted to OCR/well according to the manufacturer’s protocol. First, for internal referencing of the sensor response, the fluorescence intensity ratio (IR = I_ind_/I_ref_) was calculated for each individual well and the corresponding time point. Next, the calibration constants K100 and K0 were calculated by taking the average of IR values for wells containing cal100 (IR100) and cal0 (IR0). Oxygen concentration as a percentage of air saturation (pO_2_) for each well and measurement point was then calculated according to the following equation: pO_2_ = 100 x (K0/IR − 1)/(K0/K100 − 1). At equilibrium, when the only source of oxygen consumption is the cells at the well bottom, the rate of oxygen diffusion will equal the rate of oxygen consumption. Therefore, at steady-state conditions, the OCR per well was computed by the equation OCR = D S L ∆p/h^63^ with D = 3.3 × 10^−5^ cm^2^s^−1^ (oxygen diffusion coefficient); S = 0.348 cm^2^ (surface area exposed to the atmosphere); L = 6.0 × 10^7^ (unit conversion factor to fmol O_2_ min^−1^); h = 0.514 cm (diffusion path length, i.e., the distance between the atmosphere and the cells, which here is the depth of the medium); and ∆p = [pO_2_]_a_ − [pO_2_], where p[O_2_]_a_ is the saturated dissolved oxygen concentration at the air/media interface (~212 μM under our conditions; 37 °C, 5% CO_2_) as given by negative control wells containing MM but no cells.

### Determination of mitochondrial mass and inner mitochondrial membrane potential

As a measure of mitochondrial density/mass, protein concentration of isolated mitochondria fractions was determined and related to the cell number. The mitochondrial fractions of P40/50 and P50/70 cells were isolated using a commercial kit (Mito-Iso 2-Kit, Sigma) as described by Löhrke *et al*.^64^. Then, mitochondria were re-suspended in the storage buffer provided with the kit and the protein concentration was determined in duplicates (10 μl per sample) with Micro BCA protein assay kit (Thermo Fisher Scientific) according to the manufacturer’s instructions. The mitochondrial protein concentration (μg/ml) was related to the cell number determined before mitochondria isolation conductometrically with a Multisizer^TM^ II (Beckman Coulter). The quality of mitochondria isolation and their functional integrity were confirmed by measuring the electrochemical proton gradient of the inner mitochondrial membrane using the JC-1 assay as described by Löhrke *et al*.^64^.

### Statistical analysis

Statistical analyses were performed by using SigmaPlot 13.0 (Systat Software Inc.). Samples were tested for normality (Shapiro-Wilk) and equal variance (Brown-Forsythe). If the tests for normality and equal variances were passed, statistical significance of the data was assessed by Student’s t‐test comparing P40/50 with P50/70. If the normality test or the equal variance test was not passed, Mann-Whitney Rank Sum test was performed comparing P40/50 with P50/70. A p‐value of ≤0.05 was considered statistically significant.

## Additional Information

**How to cite this article**: Miersch, C. *et al*. Molecular and functional heterogeneity of early postnatal porcine satellite cell populations is associated with bioenergetic profile. *Sci. Rep.* **7**, 45052; doi: 10.1038/srep45052 (2017).

**Publisher's note:** Springer Nature remains neutral with regard to jurisdictional claims in published maps and institutional affiliations.

## Figures and Tables

**Table 1:** Mitochondrial mass and activity in P40/50 and P50/70 populations.

Parameter	P40/50	P50/70	p-value
Mitochondrial protein concentration (μg/ml)/10^6^ cells	62.02 ± 25.2	60.36 ± 33.0	0.948
JC-1 red fluorescence/10^6^ cells (relative fluorescence intensity)	637 ± 178	365 ± 50	0.062

Results are given as mean ± SD, n = 3. For statistical analysis Student’s t-test was performed. As a measure of mitochondrial density/mass, protein concentration of isolated mitochondria fractions was determined and related to the cell number. The functional integrity of mitochondria was assessed by measurement of the electrochemical proton gradient (Δψ_m_) of the inner mitochondrial membrane using JC-1.
